# Exercise-induced irisin in bone and systemic irisin administration reveal new regulatory mechanisms of bone metabolism

**DOI:** 10.1038/boneres.2016.56

**Published:** 2017-02-21

**Authors:** Jin Zhang, Paloma Valverde, Xiaofang Zhu, Dana Murray, Yuwei Wu, Liming Yu, Hua Jiang, Michel M Dard, Jin Huang, Zhiwei Xu, Qisheng Tu, Jake Chen

**Affiliations:** 1 Division of Oral Biology, Department of Periodontology, Tufts University School of Dental Medicine, Boston, MA 02111, USA; 2 Department of Anatomy, Research Center for Integrative Medicine, Guangzhou University of Chinese Medicine, Guangzhou, Guangdong Province 510405, China; 3 Department of Sciences, Center for Sciences and Biomedical Engineering, Boston, MA 02115, USA; 4 Department of Periodontology and Implant Dentistry, New York University College of Dentistry, New York, NY 10010, USA; 5 Department of Anatomy and Cell Biology, Tufts University School of Medicine and Sackler School of Graduate Biomedical Sciences, Boston, MA 02111, USA

## Abstract

Irisin is a polypeptide hormone derived from the proteolytic cleavage of fibronectin-type III domain-containing 5 (FNDC5) protein. Once released to circulation upon exercise or cold exposure, irisin stimulates browning of white adipose tissue (WAT) and uncoupling protein 1 (UCP1) expression, leading to an increase in total body energy expenditure by augmented UCP1-mediated thermogenesis. It is currently unknown whether irisin is secreted by bone upon exercise or whether it regulates bone metabolism *in vivo*. In this study, we found that 2 weeks of voluntary wheel-running exercise induced high levels of FNDC5 messenger RNA as well as FNDC5/irisin protein expression in murine bone tissues. Increased immunoreactivity due to exercise-induced FNDC5/irisin expression was detected in different regions of exercised femoral bones, including growth plate, trabecular bone, cortical bone, articular cartilage, and bone–tendon interface. Exercise also increased expression of osteogenic markers in bone and that of UCP1 in WAT, and led to bodyweight loss. Irisin intraperitoneal (IP) administration resulted in increased trabecular and cortical bone thickness and osteoblasts numbers, and concurrently induced UCP1 expression in subcutaneous WAT. Lentiviral FNDC5 IP administration increased cortical bone thickness. *In vitro* studies in bone cells revealed irisin increases osteoblastogenesis and mineralization, and inhibits receptor activator of nuclear factor-kB ligand (RANKL)-induced osteoclastogenesis. Taken together, our findings show that voluntary exercise increases irisin production in bone, and that an increase in circulating irisin levels enhances osteogenesis in mice.

## Introduction

Adipose tissue is a major endocrine organ that regulates energy balance and energy homeostasis.^1^ Two main types of adipose tissue have been identified, white adipose tissue (WAT) and brown adipose tissue (BAT).^2^ WAT functions primarily to store excess energy in the form of triglycerides, whereas BAT is involved in non-shivering thermogenesis, energy dissipation as heat and determination of insulin sensitivity.^3,4^ The oxidation of lipids in BAT is regulated by direct sympathetic nervous system stimulation via norepinephrine and is accomplished by expression of the mitochondrial protein uncoupling protein 1 (UCP1), which facilitates a proton leak across the mitochondrial membrane to release energy as heat and increase energy expenditure. WAT from certain depots contain “brite” adipocytes that upon stimulation can undergo a process referred to as “browning” where they take on characteristics of BAT, including the induction of UCP1 and the ability to dissipate energy through a thermogenic response.

Several reports support the notion that therapeutic strategies aimed at activating BAT activity or inducing WAT browning may counteract metabolic diseases such as obesity or type II diabetes (T2D), and could potentially ameliorate bone-loss-associated disorders. First, human BAT activity correlates inversely with energy metabolic impairment seen with aging, T2D, and obesity,^5^ and directly with high bone mineral density in healthy young women^6^ or with total cortical bone cross-sectional area in young children and adolescents.^7^ Second, WAT browning induction or activating BAT activity have been shown to suppress metabolic symptoms associated with obesity and T2D,^3,8^ whereas genetic ablation of BAT in rodents resulted in diet-induced obesity, diabetes, and hyperlipidemia.^4^ In agreement with potentially beneficial effects of BAT or BAT-like activity for bone health, WAT browning was shown to be potentially anabolic for the skeleton through *in vitro* studies in osteoblasts and osteocytes^9^ and reduced BAT function in *Misty* mice was characterized by impaired bone formation and increased bone resorption.^10^

Irisin is a peptide hormone of 112 amino acids that was initially described as a myokine derived from fibronectin-type III domain-containing 5 (FNDC5) proteolytic cleavage, which induces WAT browning, UCP1-mediated thermogenesis, and energy expenditure.^11,12^ Endurance exercise or muscle-specific overexpression of peroxisome proliferator-activated receptor-γ coactivator (PGC)-1α, a transcriptional coactivator of adaptive thermogenesis and mitochondrial biogenesis,^13^ were shown to stimulate FNDC5 expression in skeletal muscle and increase circulating levels of irisin.^11^ FNDC5/irisin was later described to be produced by WAT after exercise.^14^ In addition to exercise, circulatory levels of irisin can be upregulated in response to cold exposure in healthy human subjects.^15^ Detection of irisin has also been described in cardiac muscle,^16^ brain,^16,17^ and cerebrospinal fluid.^18^ Different laboratories attempting to investigate the activation of PGC1α/FNDC5/irisin signaling by exercise in skeletal muscle or WAT have obtained results that are difficult to reconcile. These conflicting results have been attributed to differences in the animal protocol, or their choice of anti-FNDC5/irisin antibodies.^11,14,19–22^ A limited number of studies have evaluated whether bone tissues can also express FNDC5/irisin and contribute to increasing irisin levels in circulation upon exercise or cold exposure.

Irisin levels in circulation have been shown to be lower than normal in patients with T2D,^23,24^ which exhibit increased risk of osteoporosis and bone fractures,^25^ as well as in subjects with previous osteoporotic fractures.^26,27^ Similar correlation was previously described for patients with T2D with adiponectin,^28^ an adipokine produced by WAT and skeletal muscle.^2,29–31^ Adiponectin exerts anti-diabetic effects partly by increasing muscle oxidative capacity and muscle insulin sensitivity and mediates anabolic effects in bone.^32,33^ In agreement with an anabolic effect for exercise-secreted irisin in the musculoskeletal system, conditioned media from primary cultures of myoblasts and myotubes established from exercised muscles, increased osteogenic differentiation of bone marrow stromal cells *in vitro*.^34^ Recombinant irisin at a cumulative weekly dose of 100 μg·kg^−1^ for 4 weeks has been shown to increase cortical bone mass and strength in mice.^35^

In this study, we demonstrate that 2 weeks of wheel-running exercise induced PGC1α, FNDC5, and irisin expression in bone. We also found intraperitoneal (IP) administration of recombinant irisin increased trabecular and cortical bone thickness and osteoblast numbers, without significantly affecting osteoclast numbers, and induced expression of UCP1 in subcutaneous WAT. Furthermore, recombinant irisin induced osteoblastogenesis and inhibited osteoclastogenesis in bone cell lines. Taken together, our findings demonstrate bone tissues express FNDC5 and irisin, and a threefold increase of irisin levels in circulation for 2 weeks can recapitulate part of the anabolic effects of exercise in the murine skeletal system.

## Materials and methods

### Recombinant irisin, lentiviral FNDC5, EGFP constructs, and FNDC5 shRNA lentivirus

Recombinant full-length irisin protein (112 amino acids, sequence 32–143 of FNDC5) was purchased from Phoenix Pharmaceuticals Inc. (Burlingame, CA, USA). Lentiviral FNDC5, control EGFP constructs, FNDC5-set short hairpin RNA (shRNA) lentivector for specific knockdown of FNDC5 expression, and the scramble control non-specific plasmids were obtained commercially from Applied Biological Materials Inc. (Richmond, BC, Canada). Equal numbers of lentiviral FNDC5, control EGFP constructs, FNDC5 shRNA lentivector or the scrambled control plus VSV-G, pRSV-REV, and pMDLg/pRRE packaging plasmids were co-transfected in HEK293T cells in 10 cm culture dishes. Viral particles were collected from the supernatants at 24, 36, 48, and 60 h after transfection. Thereafter, the virus was concentrated using PEG8000 for 4 days and adjusted with saline to a concentration of 10^9^ transducing units per ml. FNDC5-set shRNAs targeted the following sequences with over 70% knockdown of gene expression:

187: 5′-GCCATCTCTCAGCAGAAGAAGGATGTGCG-3′;

274: 5′-CTGGAGGAGGACACAGAATATATCGTCCA;

397: 5′-AAAGATGAGGTGACCATGAAGGAGATGGG;

511: 5′-CGCCAGTATGATATCATCAAGGACAACGA-3′.

### Mice, voluntary wheel running, and other *in vivo* treatments

Mice were maintained and used in accordance with recommendations from the Guide for the Care and Use of Laboratory Animals prepared by the Institute on Laboratory Animal Resources, National Research Council (Department of Health and Human Services Publication NIH 86–23, 1985) and by guidelines established by the Institutional Animal Care and Use Committee at Tufts University (Boston, MA, USA). Mice were maintained under standardized conditions with a 12 h light/12 h dark cycle and were provided food (standard laboratory diet) and water *ad libitum.*

Five-week-old male wild-type C57BL/6J mice (Jax #000664, Jackson Laboratory, Bar Harbor, ME, USA) and 5-week-old male APN-KO (Jax # 008195, Jackson Laboratory) weighing 17–20 g were randomly housed individually in empty cages (67 inch^2^; *n*=18) or in cages with a polycarbonate running wheel (10.16 cm diameter; Columbus Instruments, Columbus, OH, USA) mounted to the side of the cage for voluntary running (*n*=18). The running distance and average running speed were saved to a data file every 30 s by an automated computer monitoring system (Columbus Instruments). Mice run an average of 5 000 m per day for 2 weeks. Body weight was recorded at 0 and 2 weeks for each mouse. After 2 weeks of voluntary running exercise or normal cage activity, mice were killed.

Irisin protein (3.24 ng per mouse; *n*=6 mice) or saline (*n*=6 mice) was injected IP daily for 2 weeks, and mice were killed on the day after the last injection. Lentiviral FNDC5 or control EGFP viral particles (4×10^8^ transducing units per mouse) were delivered by IP injection and mice (*n*=5 per group) were killed 4 weeks later. FNDC5 shRNA lentivirus or scramble shRNA lentivirus (4×10^8^ transducing units per mouse) were delivered by IP and mice (*n*=5 per group) were euthanized after 2 weeks of voluntary running exercise.

### Tissue sampling and enzyme-linked immunosorbent assay for irisin

Several tissues were collected for messenger RNA (mRNA) and protein expression analyses including femur, tibia, red muscle (soleus), white muscle (gastrocnemius), and subcutaneous and epididymal WAT. Bone marrow was removed from bone tissues being used for western blot and quantitative real-time PCR (qRT-PCR) analysis. All tissue samples were then stored at −80 °C till further use.

Blood samples were collected by cardiac puncture, kept at room temperature for 30 min, and centrifuged at 3 000 g for 15 min. Serum samples were then stored at −80 °C. Serum irisin levels were detected with a commercially available enzyme-linked immunosorbent assay (ELISA) kit (#EK-067-16, Phoenix Pharmaceuticals, Inc.) that recognizes full-length irisin (1–112), following the manufacturer’s recommendations.

### Micro-computed tomography, histology, and immunohistochemical staining protocols

Trabecular bone architecture was assessed using a micro-computed tomography (μCT) system (Scanco μCT40, SCANCO Medical AG, Brüttisellen, Switzerland) as previously described.^33^ Bone samples were also processed for histology as previously described.^36^ Immunohistochemical staining of bone samples was performed as previously described.^37^ Primary antibody to detect irisin (epitope 42–112, 1:500) and FNDC5 expression by immunohistochemical staining was purchased from Phoenix Pharmaceuticals Inc. (#067-17). Digital images of stained tissues were taken with an Olympus BX53 microscope and analyzed by Spot Advanced software (Diagnostic Instruments, Sterling Heights, MI, USA). Bone cells were quantified as previously described.^38^

### Cell culture experiments

MC3T3-E1 osteoblast precursor cells (American Type Culture Collection, Manassas, VA, USA) were cultured in α-minimum essential medium supplemented with 10% (v/v) fetal bovine serum (Life Technologies, Carlsbad, CA, USA) and 1% penicillin–streptomycin, at 37 °C in 5% CO_2_. MC3T3-E1 were serum-starved overnight and then treated with 50 μg·mL^−1^ of ascorbic acid in the presence and absence of irisin for 7, 10, and 14 days. To induce mineralization, MC3T3-E1 cultures were treated with β-glycerophosphate in the presence and absence of irisin. Formation of bone nodules, used as indicative of mineralization *in vitro*, was monitored by alizarin red staining as previously described,^39^ followed by melting bone nodules with 10% (v/v) cetylpyridinium chloride and determination of absorbance at 562 nm as described.^40^

RAW264.7 osteoclast precursor cells (American Type Culture Collection) were cultured in RPMI 1640 with 10% fetal bovine serum (Life Technologies) and 1% penicillin–streptomycin, at 37° in 5% CO_2_. RAW264.7 cells were serum-starved overnight and then cultured in the presence and absence irisin and/or 50 ng·mL^−1^ RANKL (PeproTech, Rocky Hill, NJ, USA) for 1, 3, or 6 days. After 6 days, cells were fixed and stained for tartrate-resistant acid phosphatase activity using the K-ASSAY TRACP staining kit (Kamiya Biomedical Company, Tukwila, WA, USA) as previously described.^41^ Osteoclast-like cells were identified as red-stained cells with three or more nuclei and counted in four separate fields at a magnification of ×200. Data were reported as the mean number of osteoclast counted from three independent experiments.

C2C12 myoblast cells (American Type Culture Collection) were cultured in Dulbecco’s modified Eagle’s medium supplemented with 10% newborn calf serum and 1% penicillin/streptomycin at 37 °C in 5% CO_2_. For transductions experiments, C2C12 cells were cultured in six-well plates and treated with 10^8^ transducing units per well of lentiviral FNDC5 or EGFP viral particles in transduction medium (Iscove’s modified Dulbecco’s medium in 10% heat-inactivated fetal bovine serum) with 5 μg·mL^−1^ polybrene (Sigma, St. Louis, MO, USA). Three days later, cells were collected for FNDC5 mRNA expression analyses.

### Quantitative real-time PCR

Total RNA was isolated using the RNeasy Mini kit (Qiagen, Valencia, CA, USA). One microgram of total RNA was used for reverse transcription using the Two-Step RT-PCR Kit (Affymetrix, Santa Clara, CA, USA) according to the manufacturer’s protocol using SYBR Green Supermix (Affymetrix) on a Bio-Rad iQ5 thermal cycler (Bio-Rad Laboratories, Hercules, CA, USA). Differences in expression were evaluated by the comparative cycle threshold method using GAPDH as a control. The primer sequences are included in Table 1. RNA samples derived from animal studies resulted from five mice or more. qRT-PCR results were obtained from at least three experiments for each gene.

### Western blot analyses

Whole protein lysates from all tissues were prepared as previously described.^41^ Whole protein lysates from cell lines were prepared by using RIPA lysis buffer (Santa Cruz Biotechnology Inc., Dallas, Texas, USA) according to the manufacturer’s instructions. Nuclear proteins were purified using a nuclear extraction kit (EMD Millipore, Billerica, MA, USA). SDS-polyacrylamide gel electrophoresis and western blot analyses were performed as previously described.^33^ Antibodies for nuclear factor of activated T cells c1 (1:1 000) and lamin B1 (1:1 000) were from Santa Cruz Biotechnology. Antibody to detect irisin (epitope 42–112, 1:500) and FNDC5 expression was purchased from Phoenix Pharmaceuticals Inc. (#067-17). Antibodies for β-catenin (1:10 000) were from Sigma and those from P-AKT-1 (1:1 000), calcineurin (1:1 000), P-JNK (1:1 000), and β-actin (1:1 000) were from Cell Signaling (Danvers, MA, USA). The secondary antibodies were horseradish peroxidase-linked goat-anti-rabbit IgG (Santa Cruz Biotechnology). Blots were visualized using Pierce ECL chemiluminescence kit (Thermo Fisher Scientific).

### Statistics analysis

All data are shown as mean±s.d. from at least three different experiments and differences between groups analyzed using one-way analysis of variance. All statistical analyses were done using SPSS statistic 18.0 (SPSS Inc., Chicago, IL, USA) and values of *P*<0.05 were considered statistically significant.

## Results

### FNDC5/irisin and PGC1α expression are increased in murine bone upon voluntary wheel-running exercise

**Figure 1:**
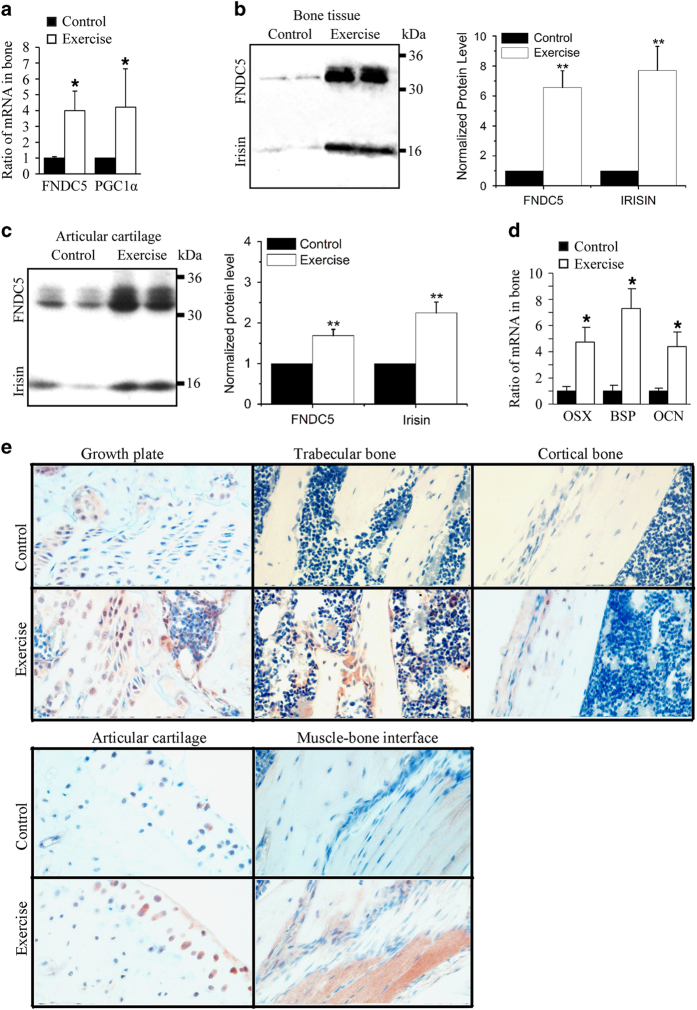
Voluntary exercise for 2 weeks increased FNDC5, PGC1α, and irisin expression in bone. Five-week-old male wild-type C57BL/6J mice weighing 17–20 g were randomly housed individually in empty cages (control group, *n*=18 mice) or in cages with a polycarbonate running wheel for voluntary running (exercise group, *n*=18 mice) for up to 2 weeks. Bone marrow was removed from bone tissues before qRT-PCR and western blot analysis. (**a**) qRT-PCR analysis of FNDC5 and PGC1-α mRNA expression in exercised and control bone tissue (*P<0.05, vs control). (**b**, **c**) Western blot analysis of FNDC5 and irisin protein expression in (**b**) exercised and control bone tissue and in (**c**) exercised and control articular cartilage (**P<0.01, vs control). (**d**) qRT-PCR analysis of osteogenic markers osterix (OSX), BSP, and osteocalcin (OCN) mRNA in bones of control and exercise group. (*P<0.05, vs control). (**e**) Representative immunohistochemistry with anti-irisin antibody reveals immunostaining in growth plate, trabecular bone, cortical bone, articular cartilage, and bone–muscle interface of control and exercised bones.

We first evaluated whether bone tissue could be a source of FNDC5 and irisin upon exercise. To that end, a group of 5-week-old mice was subjected to 2 weeks of voluntary wheel running (exercise group) and compared with a control group under routine cage activity. qRT-PCR analyses revealed that FNDC5 and PGC1α mRNA levels were increased in bone tissue from the exercise group (Figure 1a). In addition, western blot analysis demonstrated protein expression of FNDC5 and irisin increased over sixfold in bone tissue (Figure 1b) and in less extent in articular cartilage (Figure 1c) after exercise, although protein bands for FNDC5 and irisin were also detectable in control samples (Figure 1b and c).

Exercise also increased expression of markers of osteoblast differentiation in bone including osterix, bone sialoprotein, and osteocalcin (Figure 1d).

Immunohistochemical analysis of femoral bones with an antibody that recognizes FNDC5/irisin revealed increased immunostaining in the exercise group compared with the control group in different regions, including the growth plate, trabecular bone, cortical bone, articular cartilage, and muscle–bone interface (Figure 1e).

Taken together, our results demonstrated that FNDC5 and irisin are produced by bone at detectable levels by western blot analysis, and their expression is significantly increased by 2 weeks of voluntary wheel-running exercise.

### Evaluation of irisin levels in serum by ELISA

**Figure 2:**
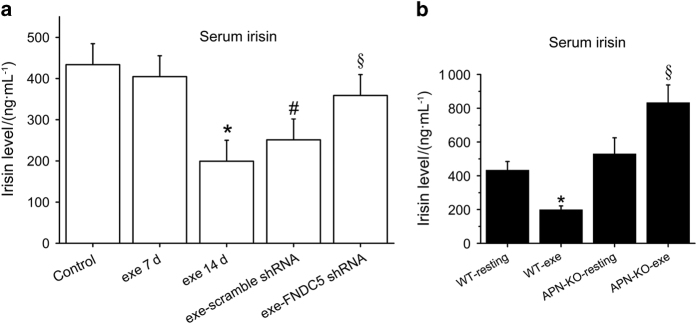
Voluntary exercise for 2 weeks increased serum irisin levels in mice lacking adiponectin expression, whereas decreased them in wild-type mice. Five-week-old male wild-type C57BL/6J mice weighing 17–20 g were randomly housed individually in empty cages (control group, *n*=18 mice) or in cages with a polycarbonate running wheel for voluntary running for 1 week (exe7d group, *n*=5 mice) and 2 weeks (exe14d group, *n*=18 mice). Five-week-old wild-type mice were injected IP with FNDC5 shRNA (exe-FNDC5 shRNA group, *n*=5 mice) or scrambled shRNA (exe-scrambled shRNA, *n*=5 mice) and then exercised for 2 weeks. (**a**) Serum levels of irisin were evaluated by ELISA in wild-type mice subjected to exercise for 7 and 14 days or subjected to FNDC5 shRNA or scramble shRNA treatments and 2 weeks of exercise. (*P<0.05, vs control; ^#^*P*<0.05 vs control; ^§^*P*<0.05 vs exe14d group). (**b**) Five-week-old male knockout mice (APN-KO) and wild-type mice (WT) were subjected to 2 weeks of voluntary wheel running (*n*=5 mice for each resting group and *n*=5 mice for each of the exercised groups). Serum irisin levels were evaluated and compared by ELISA between wild-type and and APN-KO groups. (*P<0.05, vs control; ^§^*P*<0.05 vs APN-KO-resting).

The impact of exercise on serum levels of irisin was later investigated by ELISA with an antibody recognizing the 112 amino acids of irisin in several experiments. ELISA results revealed significantly decreased serum irisin levels in mice subjected to 14 days of exercise, but levels remained unchanged as compared with the control group in mice exercised for 7 days (Figure 2a). When the 14-day exercise program was performed in mice injected with FNDC5 shRNA, irisin levels in serum were reversed to those in resting control mice, whereas plasma levels of the scrambled shRNA-treated group remained as low as for the group of mice subjected to 14 days of exercise in the absence of shRNA (Figure 2a).

Because other soluble factors, such as adiponectin, are also increased upon exercise and could be working along irisin to mediate biological effects in bone and other tissues, we determined serum levels of irisin in APN knockout mice (APN-KO) under routine cage activity and after 14 days of exercise. Our ELISA results revealed increased irisin levels in serum by exercise in mice lacking adiponectin, but not in wild-type mice of the same genetic background (Figure 2b).

### Exercise induces UCP1 expression in subcutaneous WAT and weight loss

**Figure 3:**
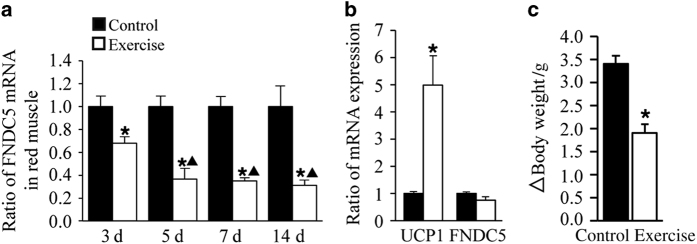
Voluntary exercise decreased FNDC5 mRNA expression in muscle, increased UCP1 mRNA in subcutaneous WAT (WAT-sub), and decreased bodyweight in mice after 2 weeks. (**a**) qRT-PCR analysis of FNDC5 expression in red muscle collected at 3, 5, 7, and 14 days from exercise and control groups. (*P<0.05 vs control; ▲*P*<0.05 vs 3 days). (**b**) qRT-PCR analysis of UCP1 and FNDC5 expression in WAT-sub at day 14 from exercise and control groups. (*P<0.05 vs control). (**c**) Exercise and control group body weight was measured on day 0 and day 14. Changes in body weight after 2 weeks are reported (*P<0.05 vs control). All values are expressed as means±s.d. (*n*=18 mice per group).

As opposed to the robust FNDC5/irisin upregulation in bone after exercise, FNDC5 mRNA expression was decreased in red muscle in the exercise group as compared with control mice (Figure 3a). Furthermore, FNDC5 mRNA expression did not change significantly in white muscle (data not shown). In addition, PGC1α mRNA expression was not affected by exercise in either red or white muscle (data not shown). UCP1 gene, a browning marker, was increased in subcutaneous WAT of the exercise group as compared with control mice, whereas FNDC5 expression did not change significantly after exercise (Figure 3b).

In agreement with an increased energy expenditure, body weight was significantly reduced in the exercise group as compared with the control group (Figure 3c). Taken together, our results suggested that exercise-induced irisin production in bone correlated with increased expression of UCP1 in WAT and decreased body weight.

### Irisin induces osteoblast differentiation, mineralization, and β-catenin protein expression in the preosteoblastic cell line MC3T3-E1

**Figure 4:**
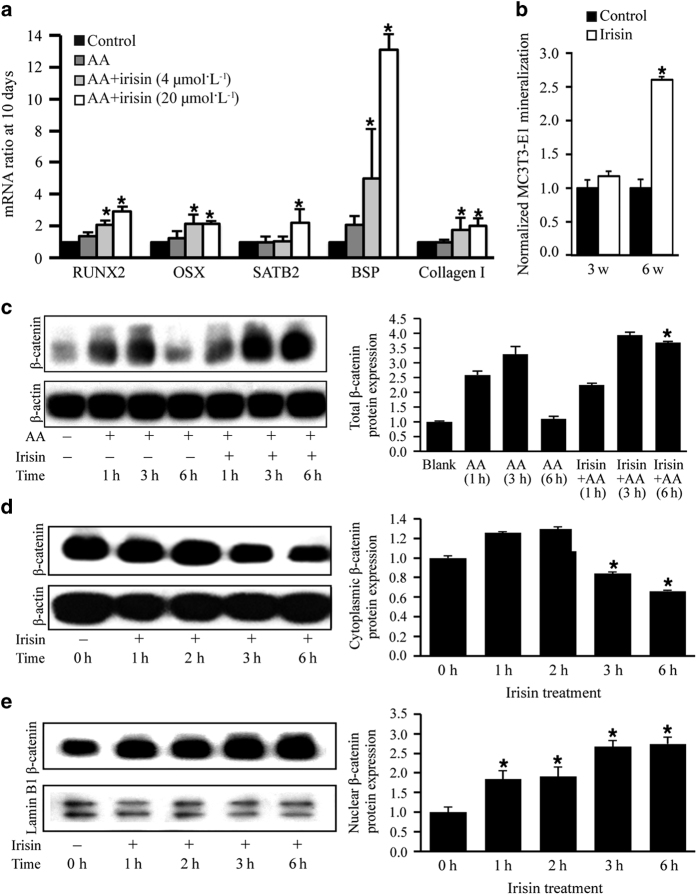
Irisin increased osteoblast differentiation and nuclear levels of β-catenin in MC3T3-E1 preosteoblastic cells. (**a**) Irisin increased ascorbic acid (AA)-induced osteoblast differentiation genes Runt-related transcription factor 2 (RUNX2), osterix (OSX), and special AT-rich sequence-binding protein 2 (SATB2) expression, and extracellular matrix proteins bone sialoprotein (BSP) and collagen I at 10 days. (*P<0.05, vs control). (**b**) Mineralization assay of MC3T3-E1 cells cultured in the presence and absence of irisin for 3 and 6 weeks (*P<0.05, vs control). (**c**) Western blot analysis to evaluate total β-catenin protein expression in MC3T3-E1 treated in the presence of AA for 1, 3, and 6 h with or without irisin and in the absence of AA or irisin (first lane). β-Actin was used as a loading control. (**d**) Western blot analysis to evaluate β-catenin protein expression in cytoplasmic extracts of MC3T3-E1 cultures treated with irisin for 0, 1, 2, 3, and 6 h. β-Actin was used as a loading control. (**e**) Western blot analysis to evaluate β-catenin protein expression in nuclear extracts of MC3T3-E1 cultures. Lamin B1 was used as a loading control. All values are expressed as means±s.d. (*P<0.05 vs control).

We then evaluated the direct effects of recombinant irisin on osteoblast differentiation *in vitro*. When pre-osteoblastic MC3T3-E1 cells were treated with recombinant irisin, mRNA expression of several osteogenic markers was upregulated, including osterix, Runt-related transcription factor 2, special AT-rich sequence-binding protein 2, bone sialoprotein, and collagen I (Figure 4a). In addition, irisin significantly increased mineralization of MC3T3-E1 cells at 6 weeks (Figure 4b).

In agreement with irisin-activating bone formation signaling mechanisms, we detected significantly higher β-catenin protein expression in MC3T3-E1 cultures treated with irisin and ascorbic acid for 6 h than in control cells (Figure 4c). Irisin also significantly decreased levels of cytoplasmic β-catenin at 3 and 6 h (Figure 4d), whereas increased nuclear β-catenin levels (Figure 4e) at all times investigated.

Taken together, our results suggested that irisin can signal osteoblasts directly and increase osteoblastogenesis and mineralization via β-catenin signaling.

### Irisin inhibits RANKL-induced osteoclast differentiation in pre-osteoclastic RAW264.7 cells

**Figure 5:**
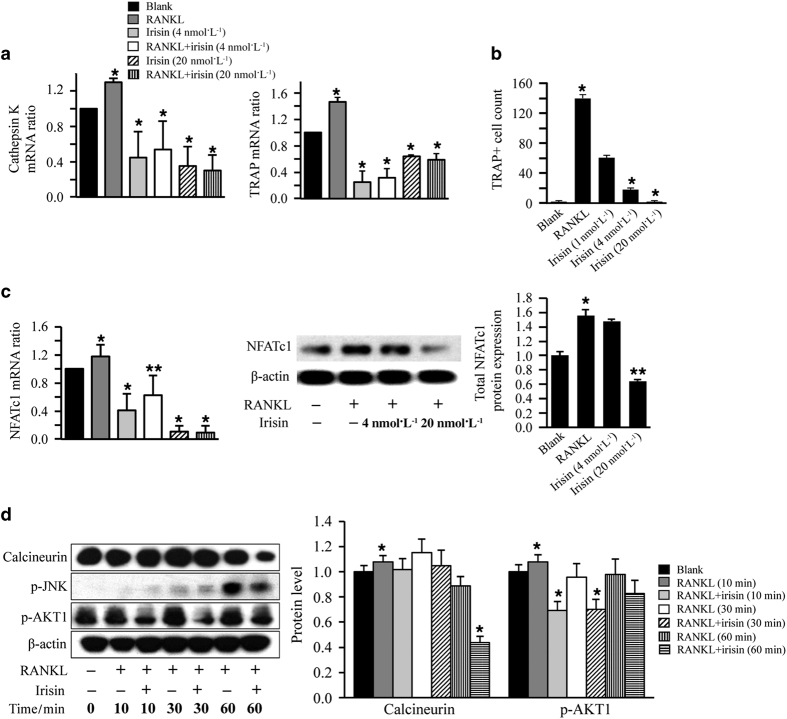
Irisin reduced RANKL-induced osteoclastogenesis by inhibiting nuclear factor of activated T cells c1 (NFATc1) expression in RANKL-treated RAW264.7 cells. (**a**) qRT-PCR analysis of osteoclast differentiation markers cathepsin K and tartrate-resistant acid phosphatase (TRAP) in RAW264.7 cells treated with RANKL and/or irisin for 3 days (*P<0.05, vs RANKL). (**b**) Irisin effects on formation of multinucleated TRAP+ cells in RAW264.7 cells untreated (blank), or treated with RANKL in the presence and absence of different concentrations of irisin for 6 days. Only TRAP-positive cells with three or more nuclei were manually counted and included in the analysis. (**c**) Irisin effects in NFATc1 mRNA expression and NFATc1 protein expression in RAW264.7 cells untreated (blank), or treated with RANKL in the presence and absence of different concentrations of irisin for 6 days. (*P<0.05, **P<0.01, vs RANKL). (**d**) Western blot image and quantification of calcineurin and p-AKT1 in RANKL-induced RAW264.7 cells treated with or without irisin for 0, 10, 30, and 60 min. β-Actin was used as a loading control. All values are expressed as means±s.d. (*P<0.05, vs RANKL).

We then evaluated the direct effects of recombinant irisin on RANKL-induced osteoclastogenesis in pre-osteoclastic RAW264.7 cells. Expression of osteoclast differentiation markers including tartrate-resistant acid phosphatase and cathepsin K, significantly decreased at 3 days by irisin treatment (Figure 5a). Furthermore, irisin treatment significantly decreased tartrate-resistant acid phosphatase-positive multinucleated cells in a dose-dependent manner (Figure 5b), and nuclear factor of activated T cells mRNA and protein expression levels in RANKL-treated RAW264.7 cells on day 6 (Figure 5c). In addition, irisin inhibited expression of calcineurin and phosphorylation of JNK at 60 min, and phosphorylation of Akt1 at 10 and 30 min in RANKL-treated RAW264.7 cells (Figure 5d).

Taken together, our results indicated that irisin significantly reduced RANKL-induced osteoclastogenesis via downregulation of nuclear factor of activated T cells c1.

### Irisin increases trabecular and cortical bone thickness *in vivo*

**Figure 6:**
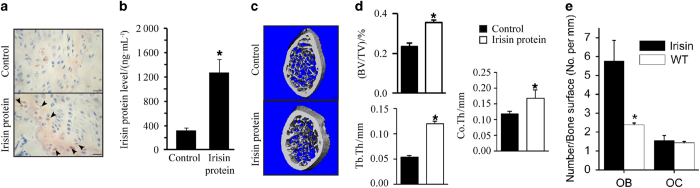
IP injection of irisin increases trabecular bone mass and osteoblast numbers in mice. (**a**) Representative immunohistochemistry with anti-FNDC5/irisin antibody illustrates positive cells in bones of control and irisin-treated mice for 14 days. FNDC5/Irisin-positive osteoblasts (arrows) were found on the edge of growth plate in irisin-treated mice. (**b**) Circulatory levels of irisin were evaluated in control and irisin-treated mice by ELISA. (*P<0.05, vs control). (**c**) Representative μCT images of the distal metaphyseal regions of femora of control and irisin-treated mice (*n*=6). Scale bars, 100 μm. (**d**) Trabecular bone volume/total volume (BV/TV), trabecular thickness (Tb.Th), and cortical thickness (Co.Th) were measured by μCT in femurs of control and irisin-treated mice. Values are means±s.d. of six mice per group (*P<0.05, vs control). (**e**) Evaluation of osteoblast (OB) and osteoclast numbers (OC) in control and irisin-treated mice. Values are means±s.d. of six mice per group (*P<0.05, vs control).

We later investigated whether recombinant irisin could directly regulate bone metabolism *in vivo*. IP injections of irisin for 14 days induced the appearance of irisin-positive osteoblasts at the edge of the growth plate (Figure 6a) and led to threefold higher irisin levels in circulation than in control mice (Figure 6b). μCT analyses of femoral bones revealed significant increases of bone volume/total volume, trabecular thickness, and cortical thickness in the irisin-treated group as compared with the saline-treated group (Figure 6c and d). The irisin-treated group was also characterized by increased osteoblast numbers (Figure 6e) and unchanged osteoclast numbers (Figure 6e) as compared with the control group.

### FNDC5 increases cortical thickness of bone *in vivo*

**Figure 7:**
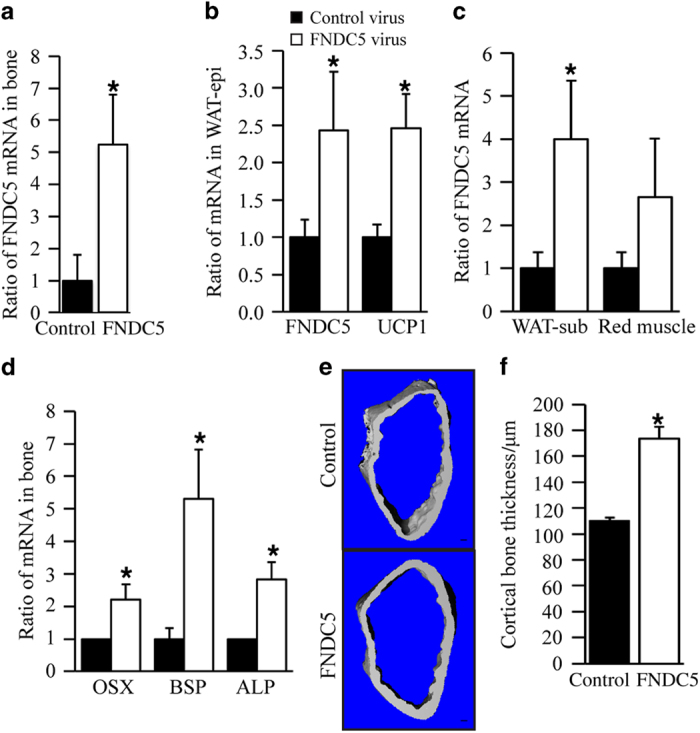
FNDC5 overexpression by IP injection of FNDC5 lentiviral particles induces expression of osteogenic markers and FNDC5 mRNA and increases cortical bone thickness. (**a**) qRT-PCR analysis of FNDC5 mRNA expression in bones of FNDC5 virus and control virus groups. (**b**) qRT-PCR analysis of FNDC5 and UCP1 mRNA expression in epididymal WAT. (**c**) qRT-PCR analysis of FNDC5 mRNA expression in subcutaneous WAT and red muscle. (**d**) qRT-PCR analysis of osteogenic markers osterix (OSX), bone sialoprotein (BSP), and ALP mRNA expression in bones (*P<0.05, vs control). (**e**) Representative μCT images of the distal metaphyseal regions of femora of control and irisin-treated mice (*n*=5). Scale bars, 100 μm. (**f**) Cortical thickness was measured by μCT in femurs of FNDC5 virus and control virus groups. Values are means±s.d. of five mice per group (*P<0.05, vs control).

Next, we used a different animal protocol in which mice were injected IP with FNDC5 or EGFP control viral particles. Four weeks after the lentiviral injections, the FNDC5 lentiviral group showed significantly increased FNDC5 mRNA expression in bone (Figure 7a). FNDC5 lentivirus injection also increased FDNC5 and UCP1 mRNA levels in epididymal WAT (Figure 7b) and subcutaneous WAT (Figure 7c). However, differences of FNDC5 mRNA levels in red muscle between control and FNDC5 lentiviral group were not statistically significant (Figure 7c). Most importantly, mRNA of osteogenic differentiation markers including osterix, bone sialoprotein, and ALP was increased in bones of the FNDC5 lentivirus group as compared with the control group (Figure 7d). μCT analyses further revealed significant increases of cortical thickness in the FNDC5 virus group as compared with the control group (Figure 7e and f).

## Discussion

In this study, we have reported that murine bone tissues express FNDC5/irisin as determined by qRT-PCR, western blot, and immunohistochemical analyses. Furthermore, we have demonstrated that 2 weeks of voluntary wheel-running exercise in mice increased FNDC5/irisin expression at the mRNA and protein levels in exercised bones as compared with controls. The exercise protocol used in our study induced PGC1α/FNDC5/irisin signaling and expression of osteogenic markers in bone, and concurrently increased UCP1 mRNA expression in subcutaneous WAT and decreased average body weight. These results were in agreement with the notion of exercise-induced irisin production by bone contributed to WAT browning by releasing irisin to circulation and increasing osteogenesis by autocrine mechanisms. Our ELISA results demonstrated that levels of irisin in circulation did not significantly change following 7 days of exercise, but significantly decreased following 14 days of voluntary running. When APN knockout mice were subjected to the same exercise protocol as wild-type mice, serum irisin levels were increased following 14 days of exercise, being the only animal model investigated in our lab that exhibited a sustained enough increase to be detected by ELISA. It is likely that adiponectin and other adipokines upregulated with exercise may work synergistically or antagonistically with irisin to mediate exercise-induced effects in bone strengthening and WAT browning. Thus, it has been reported that adiponectin is increased after 12 weeks of exercise training in humans^42^ and bone tissues express adiponectin and its receptors.^43^ Most importantly, adiponectin has been shown to promote anabolic effects in bone,^32,33^ partly by decreasing the sympathetic tone. It is also known that browning of WAT and bone remodeling are regulated by the sympathetic nervous system^10,44^ and reduced BAT function in *Misty* mice leads to altered sympathetic nervous system activity and bone loss.^10^ In conclusion, the exercise-induced irisin production in bone may not be the only mechanism leading to increased osteogenesis and WAT browning, and some of those effects could be mediated by other adipokines regulated by exercise.

Several reports in the literature have suggested or demonstrated that irisin could have a role in bone formation or bone fracture prevention. First, irisin levels in circulation were described to be lower than normal in patients with T2D,^23^ which exhibit increased risk of osteoporosis and bone fractures,^25^ and in subjects who suffered from osteoporotic fractures.^26,27^ Second, it was suggested that irisin secreted by exercised skeletal muscle could induce osteogenesis at the bone–muscle interface. In that study, authors showed that conditioned media from primary cultures of myoblasts and myotubes isolated from exercised muscles increased osteogenic differentiation of bone marrow stromal cells.^34^ Recombinant irisin was also reported to mediate anabolic effects in bone when 2-month-old mice received weekly injections of 100 μg·kg^−1^ for 4 weeks.^35^ In our study, we found mice injected at an 89-fold lower daily dose and for 2 weeks exhibited a 3-fold increase of irisin levels in circulation, which correlated with increased osteoblast numbers and enhanced mRNA expression of osteogenic markers in bone without significant effects on osteoclast numbers. Furthermore, UCP1 mRNA in WAT was upregulated, suggesting that irisin promoted the browning response as well. In agreement with the ability of recombinant irisin to recapitulate some of the beneficial effects of exercise in the skeleton by directly signaling on osteoblasts, we found irisin increased MC3T3-E1 osteoblastic differentiation and bone mineralization *in vitro.* Irisin treatment *in vivo* was shown by other investigators to decrease Sost mRNA expression^35^ and sclerostin-mediated bone response to mechanical unloading through antagonizing Wnt/β-catenin signaling.^45^ In our study, we found recombinant irisin increased nuclear localization of β-catenin in MC3T3-E1 osteoblast precursor cells *in vitro*. It is, therefore, tempting to speculate that irisin activation of Wnt/b-catenin signaling could have derived from irisin inhibiting sclerostin, which would relieve the sclerostin-mediated suppression of Wnt/β-catenin signaling, resulting in its activation. Because UCP1 mRNA was upregulated in WAT in our *in vivo* study, recombinant irisin could have mediated indirect effects on bone metabolism by promoting WAT browning, which was reported to be anabolic for the skeleton.^9,10,22^ Thus, it has been described that young women with cold-activated BAT have higher bone mineral density than those without BAT.^22^ Furthermore, impaired BAT function in *Misty* mice has been correlated with increased sympathetic tone, impaired bone formation, and increased bone resorption.^10^ Mechanistic studies have also shown that brite cells can induce osteogenesis^9^ by releasing factors including insulin-like growth factor-binding protein 2 and wingless-related mouse mammary tumor virus (MMTV) integration site 10 b that decrease sclerostin expression and increase RANKL expression by osteocytes. Because sclerostin is a negative regulator of bone formation that inhibits WNT signaling in osteoblasts,^46^ and RANKL enhances osteoclastogenesis and bone resorption, it is plausible that the indirect effects of irisin in bone metabolism through WAT browning could oppose some of the direct effects of irisin in bone cells in our *in vivo* study. Our μCT experiments demonstrated both trabecular and cortical thickness increased in the irisin-treated group respect to the saline-treated group. As overexpression of FNDC5 was previously described to induce the browning response,^11^ the increased cortical bone thickness observed in the FNDC5 lentiviral group could be partly resulting from the brown adipose tissue expansion response in addition to direct effects of cleaved irisin on bone cells. It is tempting to speculate that the bone phenotype resulting from FNDC5 lentiviral experiments mostly derived from the WAT browning response, although direct effects on osteoblast differentiation could not be discounted, as mRNA of osteogenic differentiation markers including osterix, bone sialoprotein, and ALP was increased in bones of the FNDC5 lentivirus group as compared with the control group. In a different *in vivo* study by other investigators, mice injected with an 89-fold higher daily dose of recombinant irisin for 4 weeks exhibited a significantly increased cortical bone mass and parameters of bone strength,^35^ decreased osteoclast numbers, and increased osteoblast numbers. The higher bone response of irisin in that study could be partly derived from a lack of induction of the browning response and/or higher irisin levels being induced in circulation than in our *in vivo* studies with either recombinant irisin or FNDC5 lentiviral experiments.

Different laboratories attempting to investigate the activation of PGC1α/FNDC5/irisin signaling by exercise in skeletal muscle or WAT have obtained results that are difficult to reconcile. Some of the inconsistencies have been attributed to differences in the exercise protocol, animal species, and mouse strains, or their choice of anti-FNDC5/irisin antibodies.^11,14,19–21^ Induction of FNDC5/irisin expression by PGC1α and exercise was initially described after 3 weeks of free wheel-running exercise in 12-week-old B6 mice and 10 weeks of aerobic training in non-diabetic human subjects.^11^ Other studies replicated the stimulatory effect of exercise on irisin secretion in humans^11,15,47^ and demonstrated that irisin secretion could be induced not only by exercise but also by cold exposure in healthy subjects.^15^ Additional studies using rats have suggested that there could be a good relationship between the abundance of PGC1α and FNDC5, and the oxidative capacity of skeletal muscle. Thus, it was shown that PGC1α and FNDC5 mRNA expression, and FNDC5 protein levels were not different after exercise in the soleus, but there was a significant increase in PGC1α and FNDC5 protein expression in extensor digitorum longus in high running capacity rats.^20^ Another study showed higher FNDC5 expression by rat muscle after 3 weeks of voluntary wheel exercise^14^ without a significant increase of PGC1α expression in exercised muscles. When a similar study was performed with murine muscle, mRNA expression levels of PGC1α different transcripts (resulting from alternative splicing) were slightly increased, decreased, or did not change depending on the transcript evaluated.^19^ In the same study, significant upregulation of PGC1α expression at the mRNA and protein level, was only detected after acute exercise and correlated with increased levels of irisin in skeletal muscle and serum. The same study could not detect any changes in FNDC5 protein expression after acute exercise or 3 weeks of voluntary wheel running in mouse skeletal muscle. In that study, irisin did not increase in serum or skeletal muscle after 3 weeks of voluntary exercise, but it did after a bout of acute exercise with a treadmill. In our mouse study, we found that mRNA levels were downregulated in a time-dependent manner in red muscle following 2 weeks of exercise. In addition, FNDC5 mRNA expression in WAT did not change significantly after exercise, and PGC1α mRNA was unchanged after exercise in skeletal muscle and WAT (data not shown). Another study was only able to find a transient increase of PGC1α in subcutaneous adipose tissue after a week of exercise training, but not after longer periods of exercise in rats.^14^ The transient PGC1α expression pattern in WAT correlated with increased FNDC5/irisin secretion at 1 week of exercise, and a significant decrease of FNDC5/irisin secretion at 3 weeks.^14^ Some of the inconsistencies reported by these publications and by our results can be attributed to differences in the animal protocol (different exercise program, animal species, or animal age), but also to the choice of anti-FNDC5/irisin antibodies. Thus, several studies used antibodies that could not detect irisin *per se*, but the C terminus of FNDC5 that does not include any sequence from the irisin peptide.^11,14^ However, we and others used antibodies for western blot and immunostaining that can detect a quantifiable ~12 kDa protein band due to irisin and a ~25 kDa protein band due to FNDC5 protein.^19,21^ Furthermore, we used a different antibody for ELISA experiments that was described to detect serum irisin levels specifically. Taken together, our expression analysis results accurately represent the complexity of irisin physiology, and therefore are not artifacts resulting from using the wrong anti-irisin antibodies or ELISA kits.

**Figure 8:**
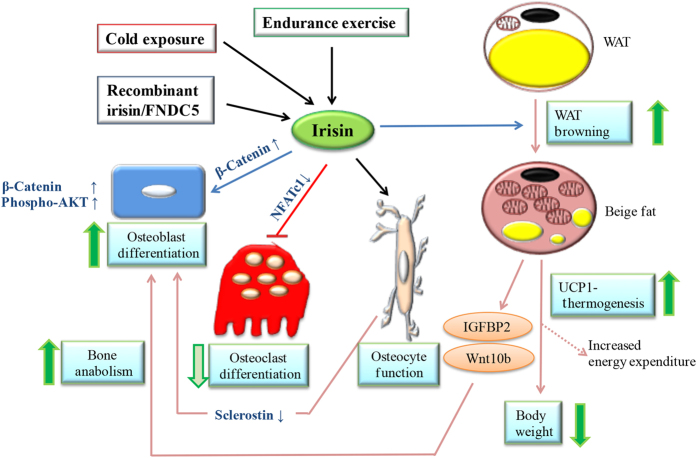
Proposed model of irisin direct and indirect effects on bone metabolism. Exercise, cold exposure, administration with recombinant irisin, or overexpressing FNDC5 can potentially lead to increased levels of irisin in circulation according to the bibliography. In our study, 2 weeks of voluntary exercise increased expression of FNDC5/Irisin and osteogenic markers in bone (Figure 1), increased serum irisin levels in mice lacking adiponectin expression (Figure 2), and upregulated UCP1 expression by subcutaneous WAT while reducing body weight (Figure 3). Recombinant irisin induced osteoblast differentiation (Figure 4) and inhibited osteoclast differentiation (Figure 5) in bone cells lines. Systemic administration of irisin (Figure 6) or FNDC5 overexpression (Figure 7) could potentially regulate bone metabolism *in vivo* by direct mechanisms on bone cells or indirectly because browning of WAT (mediated by irisin or FNDC5) is anabolic for the skeleton.^9,10,22^ Recombinant irisin has also been shown to suppress sclerostin,^35^ which mediates bone response to mechanical unloading through inhibition of the Wnt/β-catenin signaling.^45^

Our findings and the existing literature demonstrate that activation of PGC1α/FNDC5/irisin produced by bone could have a role in bone metabolism by direct and indirect mechanisms (Figure 8). Furthermore, expression of FNDC5 and irisin can be upregulated after 2 weeks of wheel-running exercise in bone, and increased irisin levels in circulation upon systemic administration *in vivo* can recapitulate part of the beneficial effects of exercise in the skeletal system. Our results also suggest that irisin may have a therapeutic potential in strengthening bone in bone-loss-associated diseases. Further experimentation will be needed to support the promise of irisin’s therapeutic potential to provide a myriad of health benefits to patients with obesity and T2D.

In conclusion, exercise-induced FNDC5/irisin may not only act as endocrine factors capable of promoting WAT browning but could also regulate bone metabolism by autocrine mechanisms. Our results provide insight into the complex regulatory interplay of muscle, bone, and fat tissues. Further experimentation will be needed to evaluate the involvement of other soluble factors increased by exercise and expressed by bone, WAT, and muscle such as adiponectin in exercise-induced irisin effects.

## Figures and Tables

**Table 1:** Primer sequences used in qRT-PCR experiments

Gene	Forward primer (5′–3′)	Reverse primer (5′–3′)
*GAPDH*	AGGTCGGTGTGAACGGATTTG	TGTAGACCATGTAGTTGAGGTCA
*FNDC5*	GAGCCCAATAACAACAAGG	GAGGATAATAAGCCCGATG
*PGC1α*	CCCTGCCATTGTTAAGACC	TGCTGCTGTTCCTGTTTTC
*UCP1*	GGCATTCAGAGGCAAATCAGCT	CAATGAACACTGCCACACCTC
*RUNX2*	AGAGTCAGATTACAGATCCAGG	TGGCTCTTCTTACTGAGAGAGG
*SATB2*	AGGCCCAAGGAATAATCAAGC	GCGTCACAACGTGATAGACATC
*OSX*	ATGGCGTCCTCTCTGGTTG	TGAAAGGTCAGCGTATGGCTT
*BSP*	CAGGGAGGCAGTGACTCTTC	AGTGTGGAAAGTGTGGCGTT
*Collagen I*	TGACTGGAAGAGCGGAGAGT	GTTCGGGCTGATGTACCAG
*ALP*	AACCCAGACACAAGCATTCC	GCCTTTGAGGTTTTTGGTCA
*OCN*	GCGCTCTGTCTCTCTGACCT	GCCGGAGTCTGTTCACTACC
*Cathepsin K*	GAAGAAGACTCACCAGAAGCAG	TCCAGGTTATGGGCAGAGATT
*NFATc1*	GGAGAGTCCGAGAATCGAGAT	TTGCAGCTAGGAAGTACGTCT

*BSP*, bone sialoproteinosterix; *NFATc1*, nuclear factor of activated T cells c1; *RUNX2*,* *Runt-related transcription factor 2; *SATB2*, special AT-rich sequence-binding protein 2.
